# Competition between the invasive macrophyte *Caulerpa taxifolia *and the seagrass *Posidonia oceanica*: contrasting strategies

**DOI:** 10.1186/1472-6785-8-20

**Published:** 2008-12-11

**Authors:** Gérard Pergent, Charles-François Boudouresque, Olivier Dumay, Christine Pergent-Martini, Sandy Wyllie-Echeverria

**Affiliations:** 1 UMR CNRS SPE 6134, University of Corsica, Faculty of Sciences, BP 52, 20250 Corte, France; 2 UMR CNRS DIMAR, University campus of Luminy, 13288 Marseilles cedex 9, France; 3 University of Washington, UW Botanic Gardens, Center for Urban Horticulture, Box 354115, Seattle, WA 98195, USA

## Abstract

**Background:**

Plant defense strategy is usually a result of trade-offs between growth and differentiation (i.e. Optimal Defense Theory – ODT, Growth Differentiation Balance hypothesis – GDB, Plant Apparency Theory – PAT). Interaction between the introduced green alga *Caulerpa taxifolia *and the endemic seagrass *Posidonia oceanica *in the Mediterranean Sea offers the opportunity to investigate the plausibility of these theories. We have accordingly investigated defense metabolite content and growth year-round, on the basis of an interaction gradient.

**Results:**

When in competition with *P. oceanica, C. taxifolia *exhibits increased frond length and decreased Caulerpenyne – CYN content (major terpene compound). In contrast, the length of *P. oceanica *leaves decreases when in competition with *C. taxifolia*. However, the turnover is faster, resulting in a reduction of leaf longevity and an increase on the number of leaves produced per year. The primary production is therefore enhanced by the presence of *C. taxifolia*. While the overall concentration of phenolic compounds does not decline, there is an increase in some phenolic compounds (including ferulic acid and a methyl 12-acetoxyricinoleate) and the density of tannin cells.

**Conclusion:**

Interference between these two species determines the reaction of both, confirming that they compete for space and/or resources. *C. taxifolia *invests in growth rather than in chemical defense, more or less matching the assumptions of the ODT and/or PAT theories. In contrast, *P. oceanica *apparently invests in defense rather than growth, as predicted by the GDB hypothesis. However, on the basis of closer scrutiny of our results, the possibility that *P. oceanica *is successful in finding a compromise between more growth and more defense cannot be ruled out.

## Background

Several theories have been advanced to explain the chemical pathways and tissue differentiation strategies that have evolved to reduce the effect of competition between different individuals of different species. Common theories proposed to explain defense strategies in plants are: Optimal Defense Theory (ODT) [1]; the Growth-Differentiation Balance Hypothesis (GDBH) [2]; the Resource Availability Theory (RAT) [3] and the Plant Apparency Theory (PAT) [4]. ODT predicts that plants should have the highest defense levels in parts that have the highest value in terms of fitness. GDBH predicts that defense allocation will be a result of trade-offs between growth (increasing plant size) and defense (or tissue differentiation); as long as all environmental factors are favorable for growth, growth processes predominate over differentiation [2]. According to RAT plants with abundant resources invest in growth rather than defense. Finally PAT is based on the observation that both types of strategy (growth and defense) occur in plants but that they differ in cost.

ODT arises from cost assumptions identified by PAT, that is that defenses are costly in terms of fitness. A further consequence is that environmentally stressed plants should be less well defended against herbivores, and therefore more palatable, than unstressed plants, as they have fewer resources available for defense [5]. Clearly, ODT-PAT assumptions (when plants are stressed, they invest in growth rather than defense) may seem incongruent with GDB-RAT assumptions (when resources are scarce, plants invest in defense rather than growth).

Patterns of plant defense and resource allocation as a function of stress, disturbance and herbivore pressure have given rise to a considerable body of literature, especially in the terrestrial realm (e.g. [2,6,5,11]). However, marine models have been relatively poorly investigated [12-15].

Interaction between the green alga *C. taxifolia *(Vahl) C. Agardh introduced into the Mediterranean Sea [16] and the endemic seagrass *P. oceanica *(Linnaeus) Delile offers the opportunity to investigate the reliability, incongruence and/or complementarity of the theories comparing defense, growth and competition. In addition, both species produce defense compounds (terpenes and phenolic acids, respectively; [17-19]) in such a way that interactions can be isolated and investigated. Here, we investigate defense strategies at the molecular level by evaluating the production of defense compounds (phenolic compounds in *P. oceanica *and Caulerpenyne (CYN) in *C. taxifolia*) and the influence of this production on growth over an annual growth cycle. To this end, we identified an interaction gradient, i.e. isolated populations and co-occurring populations and examined the effect of interaction on the two species. The purpose of this paper is to determine whether the fitness of either plant is compromised in the presence of the other; and if fitness is indeed compromised, whether a pattern of defense may be identified.

## Results

### Leaf and frond length

**Figure 1:**
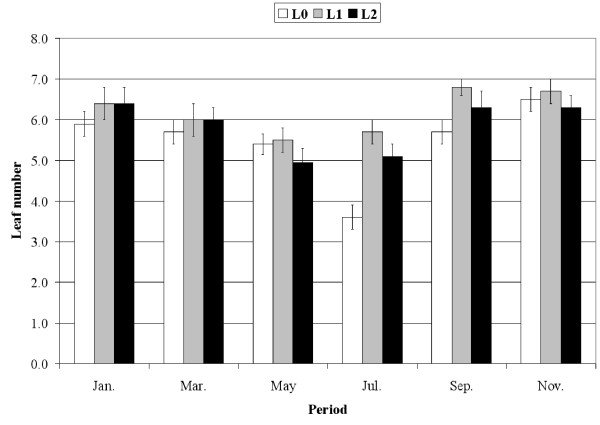
**Leaf number**. Mean number of intermediate and adult leaves of *Posidonia oceanica *according to the period and the level of interaction with *Caulerpa taxifolia*. Bars: Confidence level 95%, n = 30 shoots.

**Figure 2:**
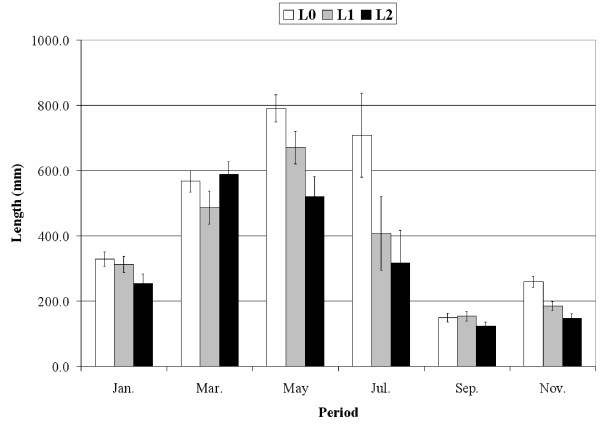
**Leaf length**. Mean length of intermediate leaves of *Posidonia oceanica *according to the period and the level of interaction with *Caulerpa taxifolia*. Bars: Confidence level 95%, n = 30 shoots.

**Figure 3:**
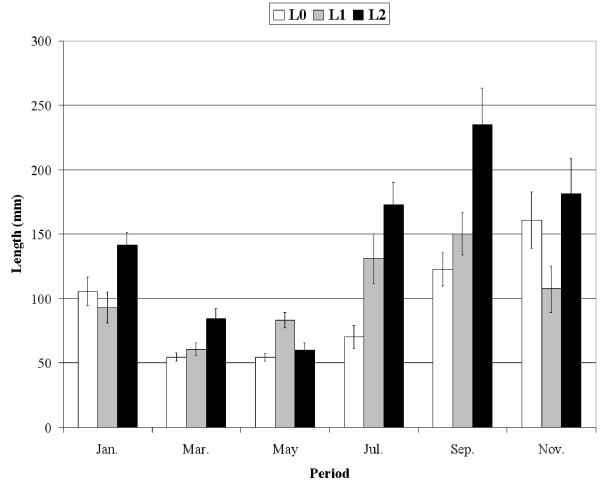
**Frond length**. Mean length of *Caulerpa taxifolia *fronds according to the period and the level of interaction with *Posidonia oceanica*. Bars: Confidence level 95%, n = 40 fronds.

*P. oceanica *shoots exhibited seasonal variation in the mean number (Fig. 1) and mean length of leaves (Fig. 2). The length of both adult (752.3 ± 43.1 mm for L0 and 509.7 ± 55.3 for L3) and intermediate leaves (Fig. 2) decreased significantly when the level of interaction with *C. taxifolia *increased (ANOVA; F = 40.1, df = 2, P < 0.001; F = 54.8, df = 2, P < 0.001, respectively). As a result, the biomass of *P. oceanica *shoots decreased from L0 (no interaction) through L2 (high interaction); for instance in May 1999, biomass is respectively, 125.3 ± 16.5, 84.6 ± 10.4 and 67.5 ± 9.4 mg dry weight, for L0, L1 and L2 (54.5, 47.8 and 38.1 g dry weight per m^2^). Similarly, the mean frond length of *C. taxifolia *changed seasonally (Fig. 3) but in the opposite direction from *P. oceanica *: the length significantly increased with the level of interaction (ANOVA; F = 89.9, df = 2, P < 0.001).

### Leaf renewal and primary production of *Posidonia oceanica*

**Figure 4:**
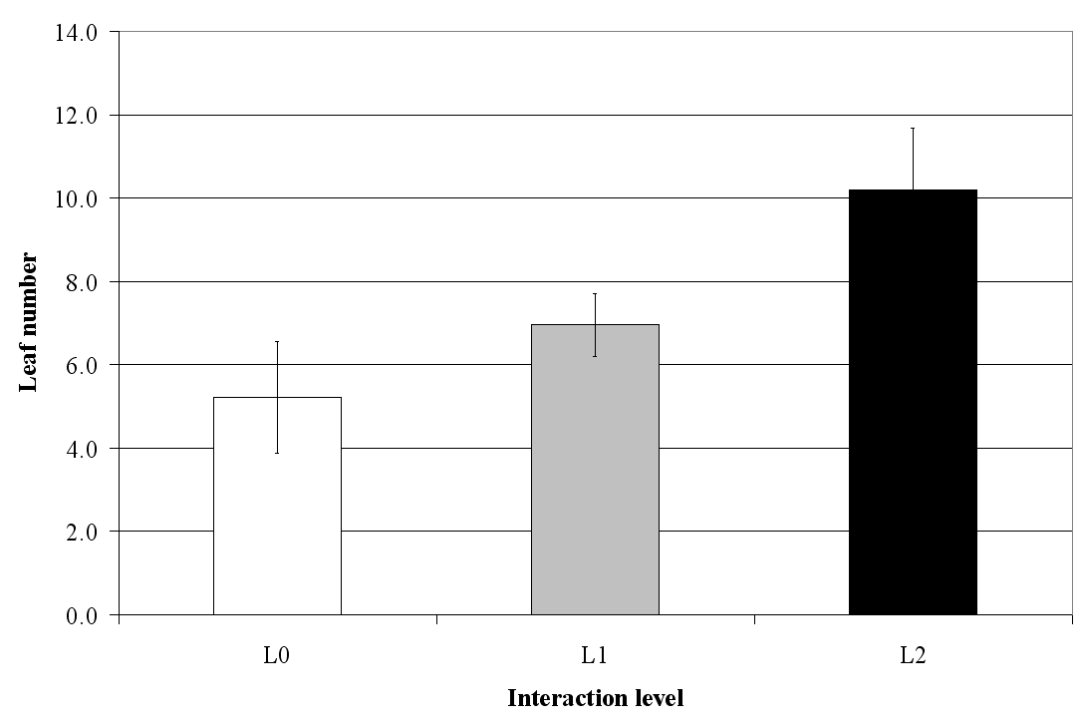
**Leaf production**. Mean number of *Posidonia oceanica *leaves produced per shoot during the year of study according to the level of interaction with *Caulerpa taxifolia*. Bars: Confidence level 95%, n = 30 shoots.

**Figure 5:**
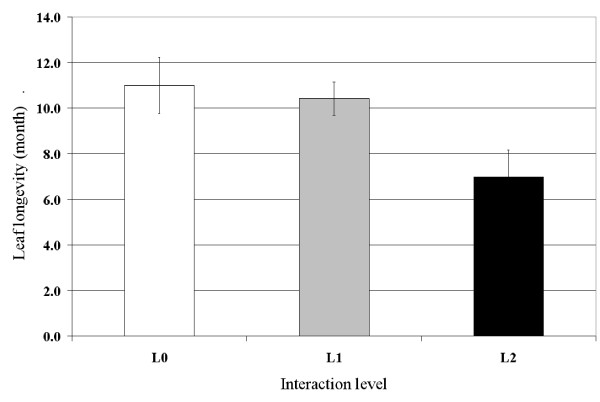
**Leaf life-span**. Mean life-span of *Posidonia oceanica *leaves, according to the level of interaction with *Caulerpa taxifolia*. Bars: Confidence level 95%, n = 5 (L0) n = 7 (L1), n = 10 (L2).

**Figure 6:**
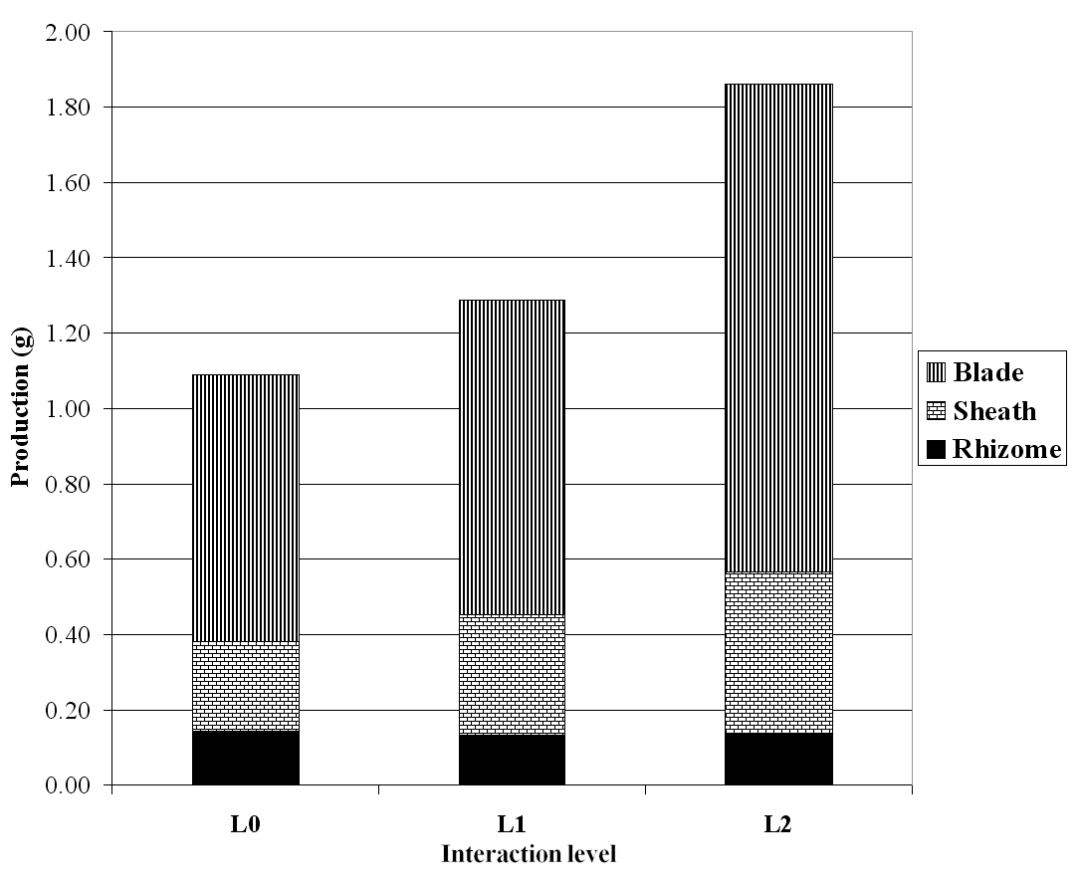
**Posidonia primary productionr**. Net primary production (in g dry weight shoot^-1 ^yr^-1^) of *Posidonia oceanica *dedicated to rhizomes, sheaths and blades, according to the level of interaction with *Caulerpa taxifolia*. The method of estimation (see [21] Pergent and Pergent-Martini, 1991) does not allow calculation of a confidence interval.

The number of *P. oceanica *leaves formed during a one-year period increased with the level of interaction (Fig. 4) while the mean life-span of leaves decreased significantly (Fig. 5; ANOVA; F = 14.4, df = 2, P < 0.001). The increase in the number of leaves produced during the study period generated an increase in the net primary production dedicated to leaf blades and sheaths (Fig. 6), with an 82% increase observed between L0 (no interaction) and L2 (highest interaction) (see Additional file 1).

### Tannin cells in *Posidonia oceanica *leaves

**Figure 7:**
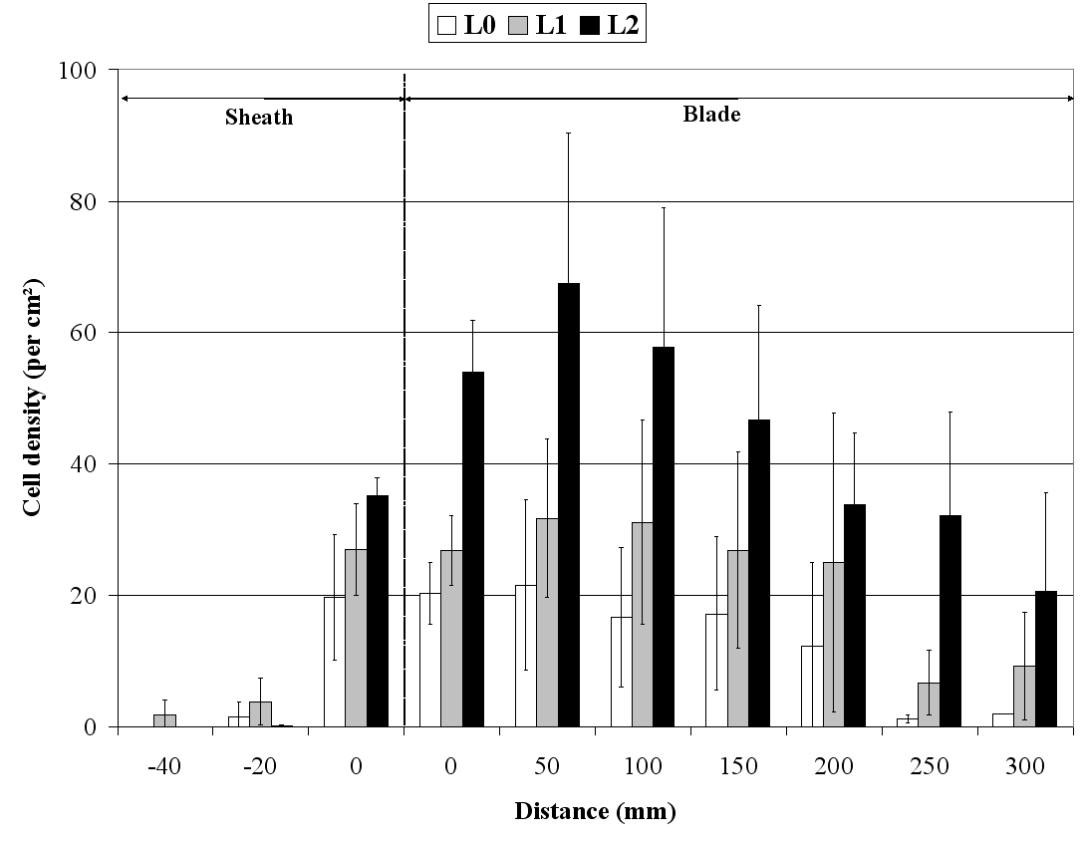
**Tanin cells**. Change in the mean density of tannin cells along the length of *Posidonia oceanica *adult leaves (sheaths and blades) as a function of the level of interaction with *Caulerpa taxifolia*. Distance along the leaf was measured below and above the limit between sheath and blade. For each adult leaf of each shoot several replicates were performed; the total number of replicates was n = 78 (L0) n = 89 (L1), n = 83 (L2). Bars: Confidence level 95%,.

The density of tannin cells varied significantly along *P. oceanica *adult leaf, with a peak in the central part of the leave (Fig. 7; ANOVA; F = 3.5, df = 7, P < 0.05). A significant increase in the density of tannin cells in blades was apparent with increasing levels of interaction with *C. taxifolia *(ANOVA; F = 29.3, df = 2, P < 0.001). For example, at 100 mm above the base of the leaf, the mean density was 16.7 ± 10.6 cells cm^-2 ^(L0), 31.1 ± 15.5 (L1) and 57.8 ± 21.2 (L2).

### Phenolic compounds of *Posidonia oceanica *leaves

Five major phenolic compounds were identified; 4-hydroxybenzoic acid, 4-coumaric acid, *trans*-cinnamic acid, caffeic acid and a mixture (hereafter P1) of at least two compounds, one of which is ferulic acid. Among minor phenolic compounds, the methyl 12-acetoxyricinoleate (hereafter P2) presented changes with the level of interaction (see below).

**Figure 8:**
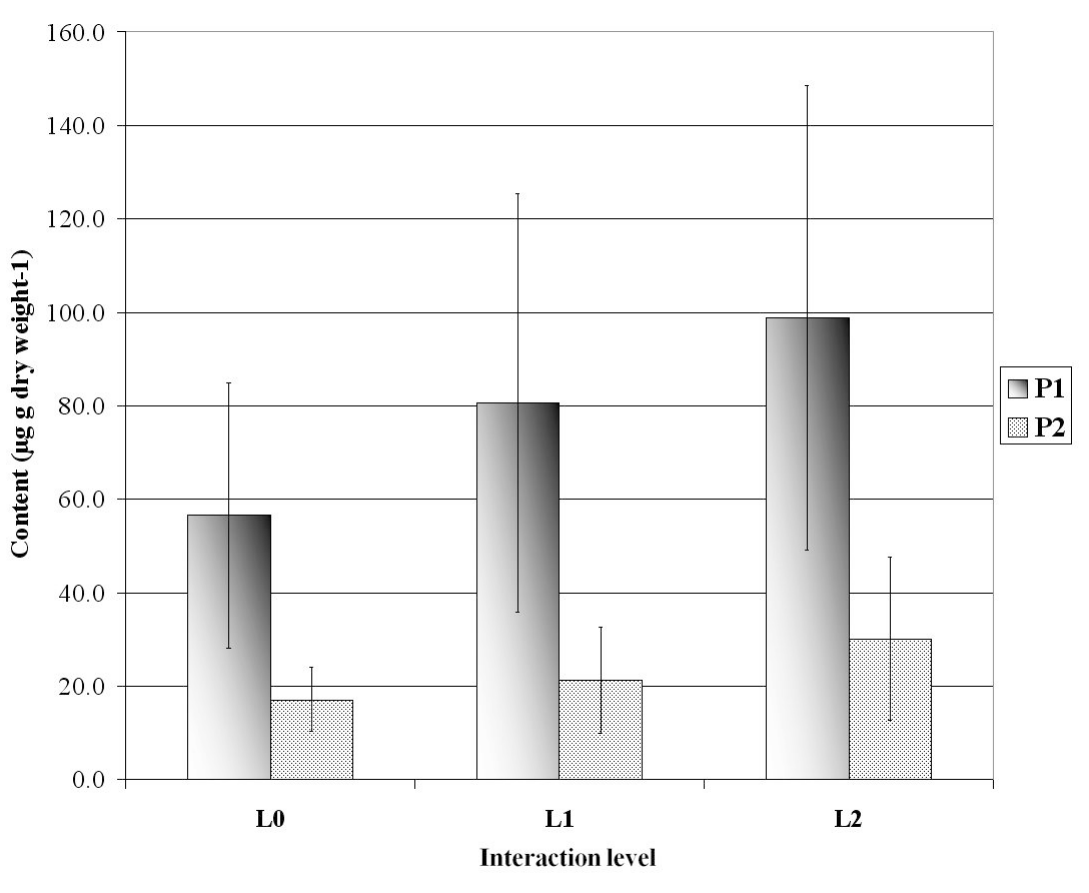
**Phenolic compounds**. Mean annual content in P1 (a mixture with ferulic acid) and P2 (methyl 12-acetoxyricinoleate) phenolic compounds in adult leaves of *Posidonia oceanica*, as a function of the level of interaction with *Caulerpa taxifolia*. Bars: Confidence level 95%, n = 17 (L0) n = 18 (L1), n = 18 (L2).

No clear seasonal trend was evident over time in the total phenolic content of *P. oceanica *leaves (data not presented). There is a weak but not significant (ANOVA; F = 2.7, df = 2, P = 0.07) increase in mean total phenolic content between L0 (297 ± 65 μg g dry weight^-1^) and L2 (357 ± 100). Conversely, P1 and P2 exhibited a significant increase with the level of interaction with *C. taxifolia *(Fig. 8; ANOVA; respectively F = 8.6, df = 2, P= 0.0009; F = 5.4, df = 2, P= 0.0091).

### Caulerpenyne (CYN) content in *Caulerpa taxifolia *fronds

**Figure 9:**
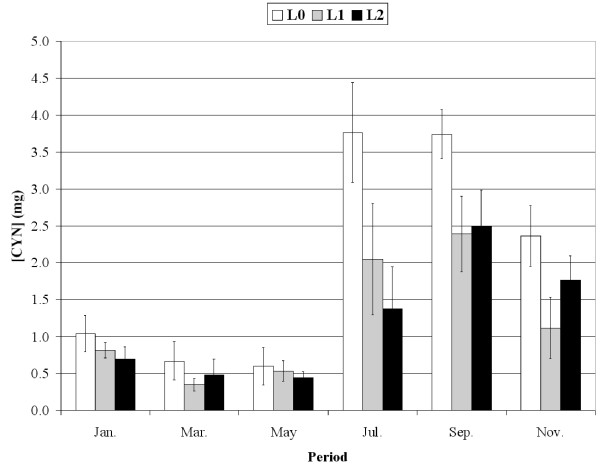
**Caulerpenyne content**. Caulerpenyne (CYN) content (in mg CYN g wet weight^-1^) in *Caulerpa taxifolia *fronds as a function of both season and level of interaction with *Posidonia oceanica*. Bars: Confidence level 95%, n = 5.

CYN content of *C. taxifolia *fronds varied seasonally (Kruskal-Wallis test; p < 0.01), with a maximum in summer and a minimum in spring (Fig. 9). Whatever the season, CYN content varied as a function of the level of interaction with *P. oceanica *(Kruskal-Wallis test; p < 0.01); in July, for instance, CYN values were 3.7 ± 0.7 mg CYN g wet weight^-1 ^(L0), 2.0 ± 0.8 (L1) and 1.4 ± 0.6 (L2).

## Discussion

### *Caulerpa taxifolia *strategy

When in competition with *P. oceanica, C. taxifolia *exhibitsincreased frond length (growth) and decreased CYN content (tissue differentiation). This may be influenced by the low levels of irradiance observed beneath the *P. oceanica *canopy (e.g., [20,21]). Increased growth is often linked to light availability. A similar competition type (for resources; see [22]) was also observed in another invasive species, *Sargassum muticum *(Yendo) Fensholt [23]. Clearly, the response of *C. taxifolia *to competition is to invest in growth rather than defense. However, it is worth noting that increased frond length does not necessarily imply an increase in primary production, because longer fronds may be slender.

Though terpenes should be considered as rather low cost defense metabolites [4,24], they do appear to be too costly for *C. taxifolia*, since the plant reaction is to lower CYN concentration. In general, terpene production, mainly CYN, defends *C. taxifolia *against herbivory [25,17-19,26] but is also essential for the wound closure of the cells [27]. For example *C. taxifolia *is avoided by herbivorous sea-urchins (*Paracentrotus lividus*) and fish (*Sarpa salpa*) [28-30]. According to [31], *C. taxifolia *is less palatable to sea-urchins than *P. oceanica *in summer, when the terpene content is maximum, whereas the reverse occurs in winter. Being more palatable when co-existing with *P. oceanica*, *C. taxifolia *couldactually be grazed more frequently. However, no conspicuous herbivore bites were observed at any time during field work. Conversely when compared with winter and spring values, the level of chemical defense in *C. taxifolia *remains relatively high in summer (Fig. 9).

We confirmed observations by [32] and [19] with the finding that the annual cycle of CYN content exhibits dramatic changes between high summer and autumn values and relatively low winter and spring concentration (Fig. 9). This cycle is coupled with the growing season of *C. taxifolia *[33]. Our finding that the actively growing summer fronds of *C. taxifolia *could be more strongly chemically defended than decaying winter fronds illustrates ODT [1].

### *Posidonia oceanica *strategy

In contrast with *C. taxifolia*, the length of *P. oceanica *leaves decreases when in competition with *C. taxifolia *(Fig. 2). However, the leaf turn-over is faster, resulting in a reduction of leaf longevity and an increase in the number of leaves produced per year. The primary production of *P. oceanica *is therefore enhanced by the presence of *C. taxifolia *(Fig. 6). [34] also observed the reduction of leaf longevity; however leaf length was either increased (adult leaves) or reduced (intermediate leaves).

As far as the production of defense metabolites is concerned, the *P. oceanica *strategy also differs from that of *C. taxifolia*. Firstly, the overall concentration of phenolic compounds does not decline. Secondly, some phenolic compounds (including ferulic acid and a methyl 12-acetoxyricinoleate) display an increase. Third, the density of tannin cells, which are specialized in the synthesis of the phenolic compounds [35,36] increases (supporting earlier findings by [34] and [37]). While terpenes are recognized mostly in anti-herbivore interactions, phenolic compounds are supposed to be involved in defense against pathogens and allelopathy by reducing the growth of competing plants [38-41]. Nevertheless, *C. taxifolia *appears not affected by the increase of these phenolic compounds.

Though superficial interpretation of our results could lead to the conclusion than *P. oceanica *invests in defense rather than growth (since biomass declines), it appears that it in fact invests both in defense and growth (since primary production actually increases).

Another explanation for this energy imbalance may relate to the structure of the *P. oceanica *meadow and the production of below ground rhizomes. These rhizomes constitute a storage organ for nutrients and polysaccharids [42-44], as well as a route for the transfer of photosynthate between shoots [45,46]. However, the translocation hypothesis would only work for site L1, with photosynthates possibly provided by shoots located in the inner meadow. As far as site L2 is concerned, the surface area of the meadow colonized by *C. taxifolia *is much larger than the maximum distance of translocation (a few tens of centimetres), which could invalidate the hypothesis.

## Conclusion

Like many invasive species, *C. taxifolia *is able to quickly colonize open areas and synthesize defense metabolites, namely terpene compounds, that are cheap to produce but with high turnover rates [33]. Conversely, *P. oceanica *grows very slowly [47], is a late successional species [48] and synthesizes defense phenolic compounds, that are costly to produce but more economical over time.

**Figure 10:**
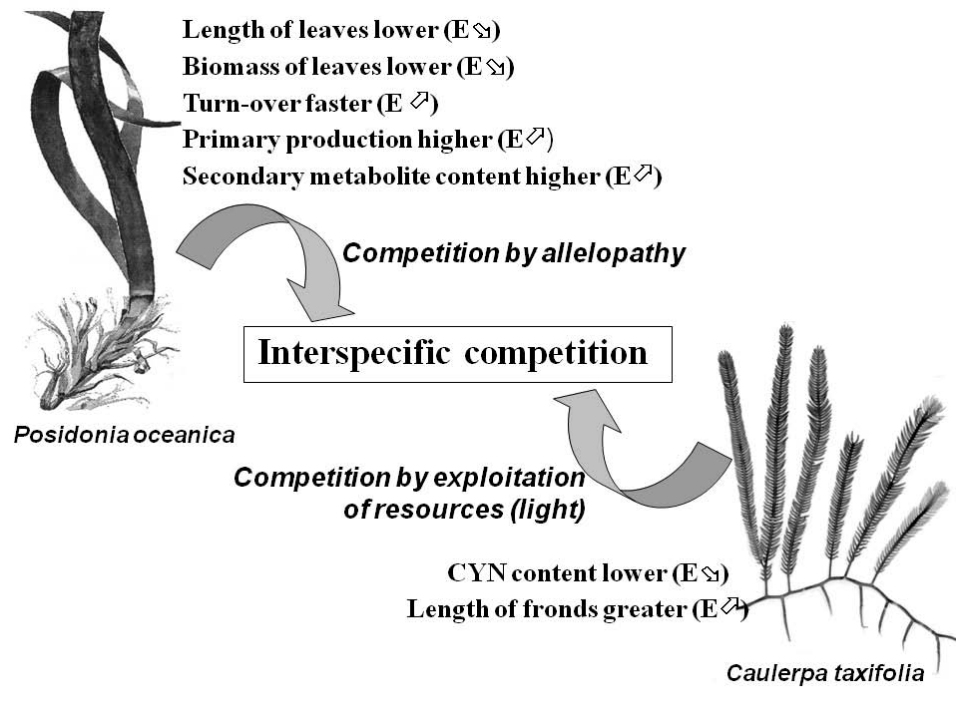
**Energy allocation and strategy**. Different energy allocations and competition strategy between *Posidonia oceanica (high left) *and *Caulerpa taxifolia *(down right). (E⇗): Increase in energy needs; (E⇘): Decrease in energy needs.

Interference between these two species determines the reaction of both (Fig. 10), which demonstrates that they compete for space and/or resources. *C. taxifolia *invests in growth rather than in chemical defense, more or less matching the assumptions of ODT and/or PAT. It is worth noting that the terpenes this plant produces are efficient against herbivores, but probably inefficient against competition with other plants, due to low water-solubility and rapid degradation in sea water [19,49]. Though investing in growth, *C. taxifolia *fronds are unable to grow taller than *P. oceanica *leaves (Fig. 2 and 3). This suggests that, at this study site, *C. taxifola *cannot successfully compete with *P. oceanica *for light. In contrast, *P. oceanica *apparently invests in defense rather than growth, as predicted by the GDB hypothesis [2]. However, we cannot rule out the possibility that *P. oceanica *may be successful in combining growth and defense.

## Methods

### Study Site

Three adjacent sites (around 10 000 m^2 ^each), situated in the subtidal region at Cap Martin (French Riviera) from 9 to 11 m, presenting similar environmental conditions (substrate, exposure, depth), were sampled every two months, from May 1999 to July 2000 using SCUBA diving. Three levels of interaction between *C. taxifolia *and *P. oceanica *were identified and replicate sampling was performed in each interaction category (Additional file 1). Formation of the *P. oceanica *meadow primarily resulted from the growth of orthotropic (erect) shoots. No seedling recruitment was observed during the course of the study. Shoot density differences were not significant between monospecific stands L0 (435 ± 64 shoots.m^-2^) and locations with co-occurring populations of *P. oceanica *and *C. taxifolia *fronds (565 ± 158). The density of *C. taxifolia *fronds was also similar. Within each site we collected randomly 30 individual sterile adult shoots of *P. oceanica *with intact rhizomes and 40 fronds of *C. taxifolia *connected to different stolons.

### Sample Processing of individual shoots and fronds

Leaf lengths were measured and the number of adult (oldest external leaves with a sheath) and intermediate leaves (younger internal leaves without sheath) was recorded according to Giraud' method [50]. Dry weights for leaf blades and leaf sheaths were computed separately. Lepidochronological analysis was also carried out to establish the average cycle of leaf renewal and estimate the annual production of leaves and rhizomes [51]. In this method, the mean leaf primary production corresponds to the mean number of leaves produced per shoot and per year multiplied by sheath and blade biomass. The length of *C. taxifolia *fronds was measured to the nearest millimeter.

### Tannin cells analysis

In September 1999, a sub-sample of three shoots of *P. oceanica *was preserved in ethanol (ethanol – 95%), to observe tannin cells. Once rinsed with fresh water, transverse sections (50 μm thick) were performed along the adult leaves at 2 cm intervals (sheath) and 5 cm intervals (blade). Tannin cells were then counted using optical transmission microscopy, enlargement ×100 and density expressed as a number of cells cm^2^.

### Preparation and chromatographic analysis of phenolic compounds

*P. oceanica *shoots were kept at low temperatures (1–5°C) during transport. In the laboratory, leaf epiphytes were removed using a razor blade and leaves were freeze dried for 72 hours (HETO^®^, Lab Equipment-FD4). Extraction of phenolic compounds was then initiated [52,38]. 1 to 3 g dry weight of leaf tissues were infused for 3 h in 200 ml of aqueous ethanol (1:1), in darkness (40°C). Extraction was carried out with ethyl acetate after vacuum evaporation of the ethanol, at 45°C. The organic phase was thus separated in the separatory funnel, dried using anhydrous sodium sulphate, and then evaporated until a dry residue was obtained. The liposoluble phenolic compounds extracted were stabilized by conversion of the hydroxyl groups into trimethylsilyl groups and the dry extract was added to 50 μl of the mixture trimethylchlorosilane: hexamethyldisilazane: pyridine (1:3:9), 100 μl of bis(trimethylsilyl)trifluoroacetamide and 1.5 μl of trimethylchlorosilane and heated at 70°C for 30 minutes in an inert atmosphere.

For each interaction level, three stabilized samples were analysed by GC and by GC-MS. The GC analyses were carried out using a Perkin-Elmer Autosystem GC apparatus equipped with FID detectors and fused-silica capillary column (30 m × 0.25 mm i.d., film thickness 0.25 μm), Rtx-1 (dimethyl polysiloxane). Oven temperature was programmed to increase the temperature environment by 2°C/m increments between 60°C to 230°C and then hold temperature at 230°C for 35 min. Injector and detector temperatures were maintained at 280°C. Samples were injected in the split mode (1:80), using helium as a carrier gas (1 ml/min).

The GC-MS analyses were performed on a Perkin-Elmer quadrupole MS system (model Q-mass 910). MS conditions occurred as follows: ionisation voltage of 70 eV, scan rate 1 scan/s, mass range 35–350 Da, ion source temperature 200°C. The spectrometer was directly coupled to a Perkin-Elmer Autosystem GC. A fused-silica capillary column (30 m × 0.25 mm i.d., film thickness 0.25 μm), Rtx-1 (dimethyl polysiloxane) was employed. The temperature conditions and the carrier gas were the same as above.

Compound identification was based on: *(i) *comparison between retention times on an apolar column, and those of standards injected beforehand, and *(ii) *computer matching with commercial mass spectra libraries [53]. A standard curve derived from pure products enabled the concentrations of phenolic compounds in the samples to be quantified.

### Preparation and chromatographic analysis of Caulerpenyne (CYN)

Five samples of *C. taxifolia *were taken from each experimental site. Algal fronds were processed by rinsing in fresh water, storing at -20°C in MeOH at a concentration of 5 g wet weight of each frond in 50 ml of MeOH (MeOH, 95–98%, Chromanorm; HPLC quality), in order to avoid any degradation of the CYN. Extraction of CYN, the major terpene compound produced by *C. taxifolia *[19], was performed directly in the MeOH. To ensure the total diffusion of the CYN present within each frond, samples were sonicated for five minutes. CYN measurements were performed using High Performance Liquid Chromatography. Thus, 10 μl of each sample was injected into the glass column of 5 μm silica (100 × 3 mm, Chrompack) and eluted with a MeOH – water solvent mixture (8:2) at a speed of 0.5 ml min^-1^. As the retention time of CYN is of the order of 2.8 min, the injection time for each sample was set at 6 min. Measurements were performed at a UV wavelength of 254 nm, sensitivity = 0.32. The HPLC pump (Waters 600), equipped with an automatic injector (Waters 717), was monitored using specially-designed software (Millennium Waters software), that also controlled the PAD data acquisition (Photodiode Array Detector Waters 996). This system enables CYN peaks to be identified during elution both in real time and under the measurement conditions. Three replicates were performed for each sample to assess the analytical dispersion. The standard curve, established on the basis of purified CYN, allowed determination of CYN levels in the samples. A direct relationship between HPLC peaks and CYN levels was obtained.

### Statistics

After checking for normality (Shapiro-Wilks test) and homogeneity of the variances (Bartlett's test), analysis of variance (ANOVA) was carried out using Statgraphic v.3.0 software. The factors were represented by season, the station and tissue type (adult sheaths and blades and intermediate leaves) and in the case of tannin cells, by the position of the section on the leaf. These ANOVA were then completed by a Tukey's multiple range test, in order to locate the differences. It should be noted that because of the small sample size for the study of the phenolic compounds (n = 3), the normality of the data could not be determined. However, ANOVA is a robust test under the conditions of application [54]. For each test, the null hypothesis was rejected with a probability of 95%.

## Authors' contributions

GP director of thesis (OD), co-written the paper with CFB, participated to field mission. CFB co-written the paper. OD carried out field mission and analysis. CPM responsible of the program. SWE participated to the redaction and corrected the manuscript.

All authors read and approved the final manuscript

## Supplementary Material

Additional File 1 **Table 1 – Levels of interaction**. Description of the three levels of interaction between *C. taxifolia *and *P. oceanica*. Click here for file

## References

[B1] McKey D, Rosenthal GA, Janzen DH (1979). The distribution of secondary compounds within plants. Herbivores: their interactions with secondary plant metabolites.

[B2] Lorio PL (1986). Growth-differentiation balance: a basis for understanding southern pine beetle-tree interactions. Forest Ecology and Management.

[B3] Coley PD, Bryant JP, Stuart Chapin F (1985). Resource availability and plant anti-herbivore defense. Science.

[B4] Fenny P (1976). Plant apparency and chemical defense. Recent Advances in Phytochemistry.

[B5] Elger A, Barrat-Segretain MH, Amoros C (2002). Plant palatability and disturbance level in aquatic habitats: an experimental approach using the snail *Lymnaea stagnalis*. Freshwater Biology.

[B6] Herms DA, Mattson WJ (1992). The dilemma of plants – to grow or defend. Quaterly Review of Biology.

[B7] Yamamura N, Tsuji N (1995). Optimal strategy of plant antiherbivore defense- implications for apparency and resource-availability theories. Ecological Research.

[B8] Hyvärinen M, Koopmann R, Hormi O, Tuomi J (2000). Phenols in reproductive and somatic structures of lichens: a case of optimal defence?. Oikos.

[B9] Ohnmeiss TE, Baldwin IT (2000). Optimal Defense Theory predicts the ontogeny of an induced nicotine defense. Ecology.

[B10] Siemens DH, Garner SH, Mitchell-Olds T, Callaway RM (2002). Cost of defense in the context of plant competition: *Brassica rapa *may grow and defend. Ecology.

[B11] Stamp N (2003). Out of the quagmire of plant defense hypotheses. Quarterly Review of Biology.

[B12] Steinberg PD (1984). Algal chemical defense against herbivores: allocation of phenolic compounds in the kelp *Alaria marginata*. Science.

[B13] Peckol P, Crane JM, Yates JL (1996). Interactive effects of inductible defense and resource availability on phlorotannins in the North Atlantic brown alga *Fucus vesiculosus*. Marine Ecology Progress Series.

[B14] Pavia H, Toth G, Aaberg P (2002). Optimal defense theory: elasticity analysis as a tool to predict intraplant variation in defenses. Ecology.

[B15] Arnold TM, Targett NM (2003). To grow and defend: lack of tradeoffs fro brown algal phlorotannins. Oikos.

[B16] Meinesz A, Belsher T, Thibaut T, Antolic B, Ben Mustapha K, Boudouresque CF, Chiaverini D, Cinelli F, Cottalorda JM, Djellouli A, El Abed A, Orestano C, Grau AM, Ivesa L, Jaklin A, Langar A, Massuti-Pascual E, Peirano A, Tunesi L, De Vaugelas J, Zavodnik N, Zuljevic A (2001). The introduced green alga *Caulerpa taxifolia *continues to spread in the Mediterranean. Biological invasions.

[B17] Guerriero A, Meinesz A, D'ambrosio M, Pietra F (1992). Isolation of toxic and potentially toxic sesqui- and monoterpenes from the tropical green seaweed *Caulerpa taxifolia *which has invaded the region of Cap Martin and Monaco. Helvetica Chimica Acta.

[B18] Guerriero A, Depentori D, D'ambrosio M, Pietra F (1995). Caulerpenyne-amine reacting system as a model for *in vivo *interactions of ecotoxicologically relevant sesquiterpenoids of the Mediterranean-adapted tropical green seaweed *Caulerpa taxifolia*. Helvetica Chimica Acta.

[B19] Amade P, Lemee R (1998). Chemical defence of the Mediterranean alga *Caulerpa taxifolia*: variations in caulerpenyne production. Aquatic Toxicology.

[B20] Carruthers TJB, Walker DI (1997). Light climate and energy flow in the seagrass canopy of *Amphibolis griffithii *(J.M. Black) den Hartog. Oecologia.

[B21] Dalla Via J, Sturmbauer C, Schönweger G, Sötz E, Mathekowitsch S, Stifter M, Reiger R (1998). Light gradients and meadow structure in *Posidonia oceanica*: ecomorphological and functional correlates. Marine Ecology Progress series.

[B22] Vila M, Sardans J (1999). Plant competition in mediterranean-type vegetation. Journal of Vegetation Science.

[B23] Staehr P-A, Pedersen M-F, Thomsen M-S, Wernberg T, Krause-Jensen D (2000). Invasion of *Sargassum muticum *in Limfjorden (Denmark) and its possible impact on the indigenous macroalgal community. Marine Ecology Progress Series.

[B24] Rhoades DF, Cates R (1976). Toward a general theory of plant anti-herbivore chemistry. Recent Advances in Phytochemistry.

[B25] Paul VJ, Littler MM, Littler DS, Fenical W (1987). Evidence for chemical defense in tropical green alga *Caulerpa ashmeadii *(Caulerpaceae: Chlorophyta): Isolation of new bioactive sesquiterpenoids. Journal Chemical Ecology.

[B26] Erickson AA, Paul VJ, van Alstyne KL, Kwiatkowski LM (2006). Palatability of Macroalgae that Use Different Types of Chemical Defenses. J Chem Ecol.

[B27] Adolph S, Jung V, Rattke J, Pohnert G (2005). Wound closure in the invasive green algal Caulerpa taxifolia by enzymatic activation of a protein cross-linker. Angew Chem Int Ed Engl.

[B28] Boudouresque CF, Lemée R, Mari X, Meinesz A (1996). The invasive alga *Caulerpa taxifolia *is not a suitable diet for the sea-urchin *Paracentrotus lividus*. Aquatic Botany.

[B29] Lemée R, Boudouresque CF, Gobert J, Malestroit P, Mari X, Meinesz A, Menager V, Ruitton S (1996). Feeding behaviour of *Paracentrotus lividus *in presence of *Caulerpa taxifolia *introduced in the Mediterranean. Oceanologica Acta.

[B30] Boudouresque CF (1997). Population dynamics of *Caulerpa taxifolia *in the Mediterranean, including the mechanisms of interspecific competition. Séminaire international "Dynamique d'espèces marines invasives: application à l'expansion de Caulerpa taxifolia en Méditerranée".

[B31] Boudouresque CF, Verlaque M, Lawrence J (2001). Ecology of *Paracentrotus lividus*. Edible sea-urchins: biology and ecology.

[B32] Amade P, Lemée R, Pesando D, Valls R, Meinesz A, Ribera MA, Ballesteros E, Boudouresque CF, Gómez A, Gravez V (1996). Variations de la production de caulerpényne dans *Caulerpa taxifolia *de Méditerranée. Second International Workshop on Caulerpa taxifolia.

[B33] Meinesz A, Benichou L, Blachier J, Komatsu T, Lemee R, Molenaar H, Mari X (1995). Variations in the structure, morphology and biomass of *Caulerpa taxifolia *in the Mediterranean Sea. Botanica marina.

[B34] De Villèle X, Verlaque M (1995). Changes and degradation in a *Posidonia oceanica *bed invaded by the introduced tropical alga *Caulerpa taxifolia *in the North Western Mediterranean. Botanica Marina.

[B35] Phouphas C (1962). Sur la présence des organites élaborateurs du tanin (taninoplastes) chez les *Posidonia oceanica *Del., et *Zostera marina *L. Comptes Rendus de l'Académie des Sciences Serie III Sciences de La Vie Life Sciences.

[B36] Tempel A-S (1982). Tannin measuring technique: a review. Journal Chemical Ecology.

[B37] Cuny P, Serve L, Jupin H, Boudouresque CF (1995). Water soluble phenolic compounds of the marine phanerogam *Posidonia oceanica *in a Mediterranean area colonised by the introduced chlorophyte *Caulerpa taxifolia*. Aquatic Botany.

[B38] Cariello L, Zanetti L (1979). Distribution of chicoric acid during leaf development of *Posidonia oceanica*. Botanica marina.

[B39] Kuo J, Mc Comb A-J, Larkum AWD, Mc Comb AJ, Sheperd SA (1989). Seagrass taxonomy, structure and development. Biology of seagrass, Aquatic Plant studies.

[B40] Taiz L, Zeiger E (1998). Plant defense. Plant physiology.

[B41] Tavares-Colpas F, Orika-Ono E, Domingos-Rodrigues J, de Souza-Passos JR (2003). Effects of some phenolic compounds on soybean seed germination and seed-borne fungi. Brazilian Archives of Biology and Technology.

[B42] Delgado O, Vidal M, Boudouresque CF, Meinesz A, Fresi E, Gravez V (1989). Phosphorus cycling in Mediterranean seagrass ecosystems: phosphorus content of vegetal tissus and sediments. Second International Workshop on Posidonia beds.

[B43] Pirc H (1989). Seasonal changes in soluble carbohydrates, starch and energy content in Mediterranean seagrasses. Marine Ecology PSZNI.

[B44] Alcoverro T, Manzanera M, Romero J (2001). Annual metabolic carbon balance of the seagrass *Posidonia oceanica*: the importance of carbohydrate reserves. Marine Ecology Progress Series.

[B45] Libes M, Boudouresque CF (1987). Uptake and long-distance transport of carbon in the marine phanerogam *Posidonia oceanica*. Marine Ecology Progress series.

[B46] Marba N, Hemminga MA, Mateo MA, Duarte CM, Mass YEM, Terrados J, Gacia E (2002). Carbon and nitrogen translocation between seagrass ramets. Marine Ecology Progress series.

[B47] Caye G (1982). Etude sur la croissance de la Posidonie, *Posidonia oceanica *(L.) Delile, formation des feuilles et croissance des tiges au cours d'une année. Téthys.

[B48] Molinier R, Picard J (1952). Recherches sur les herbiers de Phanérogames marines du littoral méditerranéen français. Annales Institut océanographique.

[B49] Amade P, Joncheray L, Loru F, Pesando D, Gravez V, Ruitton S, Boudouresque CF, Le Direac'h L, Meinesz A, Scabbia G, Verlaque M (2001). Caulerpenyne behaviour in seawater: by-products investigations. Fourth international workshop on Caulerpa taxifolia.

[B50] Giraud G (1979). Sur une méthode de mesure et de comptage des structures foliaires de *Posidonia oceanica *(Linnaeus) Delile. Bull Mus Hist Nat Marseille.

[B51] Pergent G, Pergent-Martini C (1991). Leaf renewal cycle and primary production of *Posidonia oceanica *in the bay of Lacco Ameno (Ischia, Italy) using lepidochronological analysis. Aquatic Botany.

[B52] Sauvesty A, Page F (1992). A simple for extracting plant phenolic compounds. Canadian Journal of Forest Research.

[B53] McLafferty FW, Stauffer DB (1994). Wiley Registry of Mass Spectral Data Mass Spectrometry Library Search System Bench-Top/PBM Version 310d.

[B54] Scherrer B (1984). Biostatistiques. Gaëtan Morin, Chicoutimi, Québec, Canada.

