# Supplementary material for: Competition between the invasive macrophyte Caulerpa taxifolia and the seagrass Posidonia oceanica: contrasting strategies
Source: BMC Ecol. 2008 Dec 11;8:20. doi: 10.1186/1472-6785-8-20 (PMC2621152; doi:10.1186/1472-6785-8-20)
Supplement: Additional File 1 — Table 1 – Levels of interaction. Description of the three levels of interaction between C. taxifolia and P. oceanica. [file 1472-6785-8-20-S1.doc]

| **Interaction** | **Description** | **Notation** | **Illustration** |
| --- | --- | --- | --- |
| No interaction | Isolated patches of each species | L0 |  |
| Intermediate interaction | Edges of isolated patches in contact | L1 |  |
| High interaction | Co-mingled populations | L2 |  |

Table 1. Description of the three levels of interaction between *C. taxifolia* and *P. oceanica*.
